# Patterns of chloroquine use and resistance in sub-Saharan Africa: a systematic review of household survey and molecular data

**DOI:** 10.1186/1475-2875-10-116

**Published:** 2011-05-09

**Authors:** Anne EP Frosch, Meera Venkatesan, Miriam K Laufer

**Affiliations:** 1 Center for Infectious Disease and Microbiology Translational Research, Department of Medicine, University of Minnesota, McGuire Translational Research Facility, 6th Street SE, Minneapolis, MN, USA; 2 Howard Hughes Medical Institute, University of Maryland School of Medicine, MD, USA; 3 WorldWide Antimalarial Resistance Network Molecular Module, University of Maryland School of Medicine, MD, USA; 4 Center for Vaccine Development, University of Maryland School of Medicine, 685 West Baltimore Street, Baltimore, MD, USA

## Abstract

**Background:**

As a result of widespread chloroquine and sulphadoxine-pyrimethamine (SP) resistance, 90% of sub-Saharan African countries had adopted policies of artemisinin-based combination therapy (ACT) for treatment of uncomplicated malaria by 2007. In Malawi, cessation of chloroquine use was followed by the re-emergence of chloroquine-susceptible malaria. It was expected that introduction of ACT would lead to a return in chloroquine susceptibility throughout Africa, but this has not yet widely occurred. This observation suggests that there is continuing use of ineffective anti-malarials in Africa and that persistent chloroquine-resistant malaria is due to ongoing drug pressure despite national policy changes.

**Methods:**

To estimate drug use on a national level, 2006-2007 Demographic Health Survey and Multiple Indicator Cluster Survey data from 21 African countries were analysed. Resistance data were compiled by systematic review of the published literature on the prevalence of the *Plasmodium falciparum *chloroquine resistance transporter polymorphism at codon 76, which causes chloroquine resistance.

**Results:**

Chloroquine was the most common anti-malarial used according to surveys from 14 of 21 countries analysed, predominantly in West Africa. SP was most commonly reported in two of 21 countries. Among eight countries with longitudinal molecular resistance data, the four countries where the highest proportion of children treated for fever received chloroquine (Uganda, Burkina Faso, Guinea Bissau, and Mali) also showed no significant declines in the prevalence of chloroquine-resistant infections. The three countries with low or decreasing chloroquine use among children who reported fever treatment (Malawi, Kenya, and Tanzania) had statistically significant declines in the prevalence of chloroquine resistance.

**Conclusions:**

This study demonstrates that in 2006-2007, chloroquine and SP continued to be used at high rates in many African countries. In countries reporting sustained chloroquine use, chloroquine-resistant malaria persists. In contrast, a low level of estimated chloroquine use is associated with a declining prevalence of chloroquine resistance.

## Background

Chloroquine, an inexpensive, safe and once effective anti-malarial, was a pillar of 20^th ^century malaria eradication and control efforts. Until recently, the only widely available alternative was sulphadoxine-pyrimethamine (SP). These therapies have been compromised by the spread of drug resistance, leading the World Health Organization (WHO) to recommend and almost all malaria endemic countries to implement policies endorsing the use of artemisinin-based combination therapy (ACT) as the primary treatment regimen for uncomplicated malaria. However, the 2008 World Malaria Report showed that only 3% of children with suspected malaria were treated with ACT [1], suggesting that many children were still receiving chloroquine and SP for malaria treatment. Continued use of these therapies contributes to the ongoing high malaria morbidity and mortality in sub-Saharan Africa and exerts selective pressure to maintain high rates of drug resistance in the malaria parasite population.

In the last decade, it was demonstrated that chloroquine-susceptible malaria parasites can regain predominance over chloroquine-resistant parasites after the withdrawal of drug pressure. In 1993, Malawi was the first African country to discontinue the policy of chloroquine use for uncomplicated malaria treatment. At that time, treatment failure rates were above 50% [2]. The presence of threonine at position 76 of the *Plasmodium falciparum *chloroquine resistance transporter gene (*pfcrt*), the molecular marker for chloroquine resistance [3], was present in approximately 85% of infections [4]. After 2001, the resistant *pfcrt *genotype was no longer detectable, suggesting that chloroquine-susceptible malaria had regained predominance in Malawi. In 2005, the return of treatment efficacy in Malawi was confirmed in a clinical trial that demonstrated that chloroquine had 99% efficacy for the treatment of uncomplicated malaria [5]. A decline in the chloroquine-resistant form of *pfcrt *has also been recently been documented in Kilifi, Kenya [6], where the policy of chloroquine was changed to SP in 1999 [7]. In Niger, where chloroquine use was not officially stopped until 2005, parasites carrying chloroquine-resistant genotypes have been increasing in recent years [8]. Other than these examples, little is known about longitudinal patterns of chloroquine resistance in African countries. Moreover, the relationship between objective measurements of drug pressure and trends in chloroquine susceptibility has not been described. This study tests the hypothesis that changes in the prevalence of chloroquine-resistant malaria will reflect changes in reported chloroquine use on a national level.

## Methods

### Drug use survey design and selection criteria

Drug use within each country was estimated based on publicly available data from Demographic Health Surveys (DHS) and Multiple Indicator Cluster Surveys (MICS). The DHS and MICS are nationally administered household surveys, which use two-stage cluster sampling to assess health indicators [9,10]. Within selected enumeration areas, households are randomly chosen for inclusion in the survey. All women in these households, ages 15 to 49 years, are eligible for participation. Child health modules are completed for each child of the respondent born within the last five years. Most surveys conducted in the last ten years assess the type of treatment used for children reporting fever or convulsions within the preceding two weeks.

Survey data were obtained through MeasureDHS and United Nations Children's Fund (UNICEF) for all sub-Saharan African countries that conducted DHS or MICS surveys from 2006--2007, which were the most recent data available in December 2009. Countries that were not malaria-endemic were excluded. Results from 2006-2007 surveys, as well as any data from any earlier DHS or MICS surveys conducted in that country, were included in the analysis.

### Drug resistance data identification and collection

Trends in chloroquine resistance were assessed by using the reported prevalence of the chloroquine-resistance marker *pfcrt *76T in countries that had household chloroquine use data available for at least two time points. A systematic literature review was performed in PubMed using the search terms [*country name *+ (*pfcrt *OR chloroquine resistance)] for all eligible countries. Publication dates were restricted to January 2001 through October 2009.

Although the presence of *pfcrt *76T does not correlate perfectly with treatment failure in populations [3,11], it is an indicator of the parasite's intrinsic resistance to chloroquine [12]. Therefore, prevalence of *pfcrt *76T measured at each site is used as a surrogate for the level of chloroquine-resistant parasites for the purposes of this study.

Studies with data reporting the distribution of *pfcrt *76 polymorphisms from population surveys or day zero samples from clinical trials were reviewed. Only infections reported as 'pure' *pfcrt *76T were included to improve comparability of estimates across studies. In sub-Saharan Africa, many infections are mixed, but precise assessment of allelic frequency within a single human host has not been conducted in most field studies. Additionally, genotyping methods vary in sensitivity of detecting minor alleles [13] and consequently in the ability to detect mixed infections. As a result, infections designated as 'mixed' may contain both sensitive and resistant *pfcrt *76 alleles at proportions that vary greatly across samples and studies. In contrast, samples designated as 'pure' *pfcrt *76T likely represent predominantly resistant infections that are more comparable in allelic composition. Countries with chloroquine-resistance prevalence data from at least four time points were included in the final analysis. For each study that met the criteria listed above, the prevalence of infection with exclusively chloroquine-resistant parasites was recorded along with additional information on inclusion criteria, genotyping methods and the study site. Authors were contacted for all studies in which these data could not be collected directly from the publication.

### Statistical analysis

Data were analysed in STATA 9.1 (StataCorp, College Station, Texas). For DHS and MICS data, treatment of an illness was defined as the reported use of anti-malarials, antibiotics, antipyretics, other symptomatic therapies, and traditional medicine treatments. The proportion with 95% confidence intervals of children who reported treatment among all children who had fever or convulsions was calculated, and the proportion of children who reported anti-malarial use of all children who had fever or convulsions was calculated. Proportions and 95% confidence intervals of children who received chloroquine, SP, amodiaquine, ACT, and quinine among the total number of children who had received treatment for fever or convulsions were calculated. Variability in survey design did not allow the reliable calculation of the proportion of children who took chloroquine, SP, amodiaquine, ACT, and quinine among those who received anti-malarials.

Multivariate logistic regression of chloroquine-resistant infection prevalence over time was performed for each country, controlling for study site as a proxy for the effects of geographical variation and study participant characteristics (genotyping method, cross-sectional survey versus clinical trial, age range, and symptomatic status). The regression coefficient for time (in years) was used to model the temporal trend in chloroquine resistance prevalence. The correlation between 2006-2007 estimated chloroquine use and most recent reported prevalence of *pfcrt *76T was calculated.

## Results

**Figure 1:**
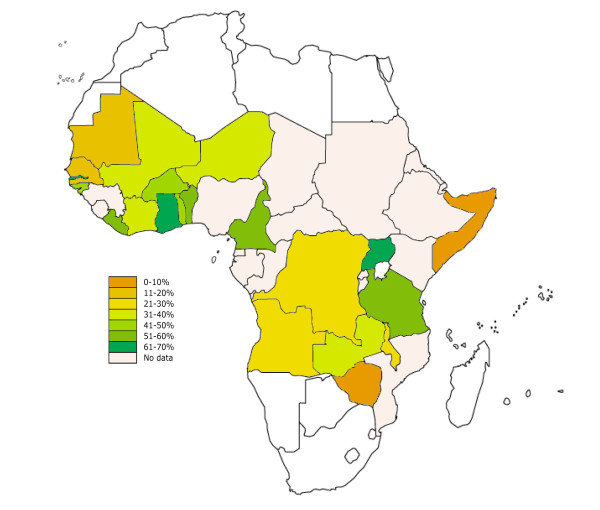
**Anti-malarial use in Africa for the treatment of fever or convulsions**. Map of African malaria endemic countries showing the proportion of children under age 5 with fever or convulsions within the last 2 weeks that reported the use of anti-malarials.

Twenty-three countries met the inclusion criteria for the drug use analysis. Twenty-one of these had data, which was publically available from the 2006--2007 survey. See Additional File 1: Supplemental Table 1 for a list of the surveys used in the analysis. The proportions of children with fever or convulsions who reported treatment with an anti-malarial are illustrated in Figure 1. The Gambia, Uganda, and Ghana had the highest proportion of children receiving anti-malarials for the treatment of fever or convulsions with 63%, 62% and 61%, respectively.

**Figure 2:**
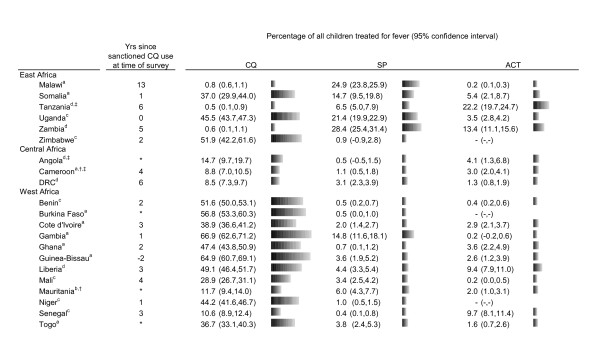
**Drug use data from 21 DHS and MICS household surveys, 2006--2007**. a MICS 2006. b MICS 2007. c DHS 2006. d DHS 2007. † >15% reporting quinine use. ‡ >15% reporting amodiaquine use. *Year of official policy change could not be confirmed.

The proportions of children who received chloroquine, SP and ACT among all children who were treated for fever or convulsions are shown in Figure 2. Chloroquine was the most common anti-malarial reported in 14 of 21 countries and reported use was particularly high in West Africa. SP was the most commonly reported anti-malarial used in two East African countries, Zambia and Malawi. Reported SP use was low throughout West Africa.

Use of anti-malarials other than SP and chloroquine was reported as relatively low across all countries analysed. Amodiaquine was the most common treatment only in Angola. More than ten percent of children also reported amodiaquine use in Tanzania, Ghana, and Cameroon. Among countries with 2006--2007 data, Tanzania was the only one in which the most commonly reported anti-malarial was an ACT, with 22% of children treated for fever reporting use of a "combination with artemisinins."

**Figure 3:**
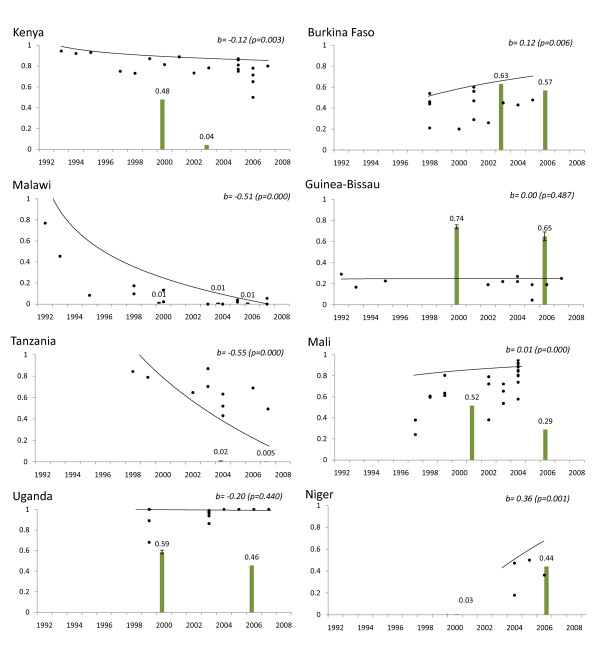
**Longitudinal chloroquine use and chloroquine resistance trends**. Bars indicate the proportion of children who had fever or convulsions in the preceding two weeks who reported chloroquine use. Observed (dots) and predicted prevalence using regression modelling (trendlines) of *pfcrt *76T are displayed with the coefficient for time in years with p-value (b) for the country regression model in the upper right hand corner of each graph.

Nineteen of the 23 countries had two or more years of DHS and MICS survey data available allowing for estimation of chloroquine use trends and were, therefore, eligible for inclusion in the analysis of the effect of reported drug use on chloroquine resistance. Among these, eight had published chloroquine resistance data covering at least four years. Molecular data spanned a range of four to 16 years per country. The studies from these countries evaluated in molecular marker analysis are described in additional file 2: Supplemental Table 2 [3-6,8,14-48]. All data from one study [49] and data from three sites in a second study [37] were excluded, both from Burkina Faso, because pure and mixed infections could not be differentiated. Reported chloroquine use and logistic regression modelling of chloroquine resistance prevalence over time are summarized in Figure 3.

Uganda, Burkina Faso and Guinea-Bissau reported >40% chloroquine use sustained across surveys, from the early to the mid-2000's. These countries did not have significant declines in the level of chloroquine resistance over time (Figure 3). However, the prevalence of chloroquine resistance among these three countries varied considerably. In Uganda, chloroquine resistance appears fixed in the population, with the prevalence of purely resistant infections at nearly 100% over a ten-year period. Chloroquine resistance in Burkina Faso increased slightly, with predicted purely resistant infection prevalence ranging from approximately 50-70%. In Guinea Bissau resistance prevalence was approximately 20%, the lowest of the three countries.

Niger was the only country among the eight countries analysed to report an increase in chloroquine use over the last decade. Based on very limited molecular surveys, the prevalence of chloroquine resistance rose, as well. The few reports of chloroquine resistance prevalence spanned a short time period, from 2003--2006.

Malawi, Kenya, Tanzania and Mali all reported low or declining chloroquine use during the study period. Reported chloroquine use was low (< 5%) in all years surveyed in Malawi and Tanzania, although chloroquine use was ubiquitous prior to the period included in this analysis. Estimated use in Kenya declined from nearly 50% to 4% between 2000 and 2003. Malawi, Tanzania and Kenya each exhibited a significant decline in the prevalence chloroquine resistance over time (Figure 3). The decline in Kenya (time coefficient -0.12), although statistically significant, is occurring at a slower rate than in Malawi or Tanzania (time coefficient -0.51 and -0.55, respectively). A decrease in but not elimination of chloroquine use between 2000 and 2006 (52% to 29%) was reported from Mali. From 1996 to 2004 there appears to have been a slight rise in the level of resistance in the sites sampled. Evidence of the distribution of drug resistance after 2006 was not available from Mali when the decline in chloroquine use was reported.

There was no correlation between most recent reported chloroquine use and *pfcrt *76T prevalence at that time point by country (slope = 0.08; 95% confidence interval = -1.21 - 1.37; r = 0.06).

## Discussion

There is a renewed commitment to pursue malaria eradication. Access to effective therapy is an essential component of this effort. With emerging concerns of artemisinin resistance in Asia [50,51] and the possibility that drugs previously compromised by resistance can regain efficacy, it is important that we elucidate the effect of drug use on the dynamics of anti-malarial resistance. A review of the most recent data on chloroquine and SP use patterns in Africa and the first multi-country analysis of the relationship between anti-malarial drug use and the prevalence of resistant infections is presented here. This review of DHS and MICS survey data reveals that, as of 2006--2007, the estimated proportion of chloroquine and SP use in many African countries remained high. Furthermore, the descriptive comparisons of reported chloroquine use to patterns in *pfcrt *supports the hypothesis that in Africa widespread chloroquine use is associated with the persistence of chloroquine-resistant parasites, while a decrease chloroquine use is associated with a decline in the resistance.

Limitations of the DHS and MICS data include inaccuracies inherent to information derived from surveys and from selection bias, arising from non-random factors influencing when and where surveys are conducted and successfully completed. Molecular marker data may also be affected by selection bias introduced in choosing study sites and by publication bias. More dense spatial representation may better estimate prevalence on a national scale, particularly because local variation in the prevalence of chloroquine resistance within the same country has been documented [33,52]. Finally, there are varying strategies to estimate haplotype frequencies within parasites causing human infection [13]. The selected method was limited to reviewing previously collected data without precise measurements of allelic frequency. Despite these limitations, the analysis presented here uses data derived from the most comprehensive sources available at this time.

### National treatment policy versus reported drug use

Of the 21 countries evaluated for their anti-malarial use in 2006-2007, only three reported highest use of the regimen that was the national first line treatment policy at the time. Among them, only Tanzania had adopted a policy of ACT use and reported survey results that corresponded with this policy. Studies that evaluate the prevalence of drug resistance may interpret their results in the context of the treatment policy at the time of the survey [52]. However, the findings presented here demonstrate the shortcomings of such an assumption. Because treatment practice may not be in accordance with the official treatment policy, realistic estimates of drug use should be included when characterizing drug pressure.

### Patterns in reported anti-malarial use

The estimated use of chloroquine and SP displays a strong regional pattern, with high chloroquine use reported in West Africa and more SP use reported in East Africa. This may be attributed to several factors. Resistance to chloroquine arose earlier and reached higher levels in East Africa [53]. As a result, treatment policies in East Africa tended to transition away from chloroquine earlier than the rest of Africa, usually with an interim policy utilizing SP. Higher levels of clinically apparent chloroquine treatment failure over a longer period of time in East Africa also may have made providers and patients more receptive to the recommended policy transition to non-chloroquine regimens. Although West Africa saw a rise in chloroquine resistance later than other areas of the continent, several studies including some reviewed in this analysis demonstrate that chloroquine-resistant parasites have come to dominate many of these parasite populations, as well [43,52,55-60].

### Chloroquine use and drug resistance

Longitudinal trends in *pfcrt *76T prevalence in Malawi, Kenya and Tanzania, the three countries with historically high but recently low chloroquine use, support the hypothesis that a decline in chloroquine resistance occurs with the withdrawal of drug pressure. In Malawi, chloroquine use was negligible by 2000 and chloroquine resistance had disappeared by 2003. Tanzania appears to follow a similar pattern, with a significant downward trend in the prevalence of *pfcrt *76T. Chloroquine resistance is declining in Kenya as well, as previously demonstrated in Kilifi [6], although more gradually than in Tanzania and Malawi. The slower rate may be a result of amodiaquine use [61], which was reported to have been taken by 9.6% of sampled children treated for fever in the 2003 DHS survey in Kenya [62]. However, reported chloroquine use was also high in Kenya until at least 2000, and the full effect of decreased chloroquine use may not become evident for several years.

The four countries with sustained reported chloroquine use at moderate to high levels exhibited no change or even slight increases in the prevalence of *pfcrt *76T. In Uganda, almost all infections were chloroquine-resistant, while in Burkina Faso and Mali half to three quarters of the infections were chloroquine-resistant. Prevalence of *pfcrt *76T in Guinea Bissau was lower than Uganda, Burkina Faso and Mali, but remained stable around 20%. The reason for these differences in prevalence is unclear. Seasonality of the transmission patterns in East versus West Africa could be a contributing factor. In Uganda, malaria transmission is perennial, characterized by year-round infections with peaks during rainy seasons. As a result, treatment occurs throughout the year, subjecting parasites to constant selective pressure. In contrast, the more seasonal transmission of the West African Sahel, including Burkina Faso and Mali, may lead to prolonged periods of lower anti-malarial use. The prevalence of chloroquine-resistant alleles was shown to increase over the transmission season and decrease between seasons in a study conducted in The Gambia, suggesting that the transient decrease in drug pressure is enough to allow for the resurgence of susceptible parasites [63].

Differences in access to anti-malarial treatment are also a possible contributor to the varying levels of *pfcrt *76T in Guinea-Bissau, Burkina Faso, Mali, and Uganda. If a large proportion of children with fever go untreated, even when chloroquine is the most commonly used anti-malarial, a large proportion of the parasite population is not subjected to chloroquine selective pressure. This may contribute to the high level of resistant parasites in Uganda, which has among the highest anti-malarial coverage for the treatment of fever, likely due to the use of home based therapies. However, this does not adequately address the differences in *pfcrt *76T levels seen in West Africa, where Guinea Bissau, Mali, and Burkina Faso had similar anti-malarial coverage for febrile and convulsive children, ranging from 33-46%. A wide range of factors including environmental conditions, access to medication, host immunity, duration of drug pressure and others likely contribute to the differing baseline levels of resistant alleles.

This study demonstrates that as of 2006-2007, reported anti-malarial use did not reflect national policy in several countries. The analysis does suggest that in Africa, changes in prevalence of *pfcrt *76T are related to estimates of drug use. This relationship raises the possibility that the level of resistance could be used as an indicator of continuing drug use. However, the lack of correlation between the most recent point estimates of drug use and prevalence of *pfcrt *76T indicates that chloroquine resistance is not always related to current levels of drug pressure. This point is exemplified by Kenya, where reported use in 2003 was 4% but *pfcrt *76T prevalence was 80% as of 2007. Rather, it appears that chloroquine resistance declines only under sustained conditions of very low or near-absent use, as demonstrated by the cases of Malawi and Tanzania. The level of resistance maintained by high drug pressure may vary by country and epidemiological situation, as seen by comparing Uganda and Guinea-Bissau. Additionally, while *pfcrt *76T has declined in some African countries, the resistant genotype remains fixed in some regions of Southeast Asia and Latin America after decades of little to no chloroquine use for *P. falciparum *infection [64-66]. This may be due to reduced parasite diversity in low compared to high transmission settings, leading to decreased opportunities for recombination and the fixation of alleles in the population. As ACT uptake increases and transmission decreases in Africa, it will be informative to examine the dynamics of chloroquine susceptibility within the parasite populations in different settings. The return of chloroquine sensitivity in Malawi has been attributed to the re-expansion of sensitive parasites that survived in the population despite drug pressure rather than reversion of resistant parasites [67]. The return of chloroquine sensitivity to other countries is likely to depend on the availability of circulating chloroquine-susceptible parasites to out-compete the resistant population in the absence of drug pressure.

The wealth of standardized information collected in DHS and MICS surveys along with published information on the prevalence of chloroquine resistance has allowed us to take a step toward understanding the relationship between estimates of drug pressure and the dynamics of drug-resistant malaria. As an increasing amount of data on molecular markers, *in vitro *and clinical estimates of resistance are made available via initiatives such as the WorldWide Antimalarial Resistance Network (WWARN), our ability to characterize the level of resistance in different geographic areas will become more comprehensive and precise. This knowledge will inform targeted, rational approaches to the study of drug resistance and the design of more efficient and cost effective programs to curb resistance.

## Conclusions

The results presented here suggest that the evolutionary effects of anti-malarial treatment pressure on malaria parasite populations can be reversed. As ACT use increases throughout Africa, it is possible that chloroquine may be used in the future with appropriate partner drugs and possibly limited to targeted populations. Moreover, as resistance to artemisinins continues to evolve in Southeast Asia, success in preventing the spread of anti-malarial resistance will depend on the ability to anticipate its emergence and spread under a variety environmental and policy scenarios.

## Competing interests

The authors declare that they have no competing interests.

## Authors' contributions

AF, MV and MKL designed the study. AF, MV collected and analysed the data. AF wrote the first draft of the manuscript. MV and MKL edited and revised the manuscript. All authors read and approved the final manuscript.

## Supplementary Material

Additional File 1 **Supplemental Table 1 Surveys used in analysis of the treatment of fever or convulsions in children under 5 years of age**. *Sample sizes taken from DHS and MICS child datasets, which include all children from sampled households under the age of 5 years. Click here for file

Additional File 2 **Supplemental Table 2: *Plasmodium falciparum pfcrt *76T prevalence studies used in chloroquine resistance prevalence analysis**. UK = unknown. m = months. y = years. UC = uncomplicated malaria. AS = asymptomatic infection. SM = severe malaria. SI = symptomatic infection. Click here for file
