# Supplementary material for: Patterns of chloroquine use and resistance in sub-Saharan Africa: a systematic review of household survey and molecular data
Source: Malar J. 2011 May 9;10:116. doi: 10.1186/1475-2875-10-116 (PMC3112453; doi:10.1186/1475-2875-10-116)
Supplement: Additional File 1 — Supplemental Table 1 Surveys used in analysis of the treatment of fever or convulsions in children under 5 years of age. *Sample sizes taken from DHS and MICS child datasets, which include all children from sampled households under the age of 5 years. [file 1475-2875-10-116-S1.PDF]

Country	Survey	Year	Total sample size*	Sample size* of children with fever in last 2 weeks
**East Africa**				
Kenya	DHS	2003	5949	2215
	MICS	2000	7266	1137
Malawi	MICS	2006	23238	8057
	DHS	2004	10914	3702
	DHS	2000	11926	4245
Somalia	MICS	2006	6373	1288
Tanzania	DHS	2007	7502	1200
	DHS	2004	8564	2094
Uganda	DHS	2006	8369	3091
	DHS	2000	7113	2628
Zambia	DHS	2007	6401	1034
Zimbabwe	DHS	2006	3643	794
**Central Africa**				
Angola	DHS	2007	1698	265
Cameroon	MICS	2006	6495	1154
DRC	DHS	2007	8992	2556

Country	Survey	Year	Total sample size*	Sample size* of children with fever in last 2 weeks
**West Africa**				
Benin	DHS	2006	16075	4204
Burkina Faso	MICS	2006	5677	1051
	DHS	2003	10645	3427
Cote d'Ivoire	MICS	2006	8604	2211
Gambia	MICS	2006	6641	538
Ghana	MICS	2006	3545	796
Guinea-Bissau	MICS	2006	6570	787
	MICS	2000	5853	2523
Liberia	DHS	2007	5799	1673
Mali	DHS	2006	14238	2094
	DHS	2001	13097	3270
Mauritania	MICS	2007	8981	1445
Niger	DHS	2006	9193	2170
	MICS	2000	5080	1902
Senegal	DHS	2006	4808	1734
Togo	MICS	2006	4154	766

Supplemental table 1: Drug use surveys

*Sample sizes taken from child data set which includes all children from sampled households under the age of 5 years.
