# Supplementary material for: Patterns of chloroquine use and resistance in sub-Saharan Africa: a systematic review of household survey and molecular data
Source: Malar J. 2011 May 9;10:116. doi: 10.1186/1475-2875-10-116 (PMC3112453; doi:10.1186/1475-2875-10-116)
Supplement: Additional File 2 — Supplemental Table 2: Plasmodium falciparum pfcrt 76T prevalence studies used in chloroquine resistance prevalence analysis. UK = unknown. m = months. y = years. UC = uncomplicated malaria. AS = asymptomatic infection. SM = severe malaria. SI = symptomatic infection. [file 1475-2875-10-116-S2.PDF]

Country	Site	Year(s)	Age range	Malaria status	Sample size
**East Africa**					
Kenya	Kilifi [6]	1993	6 - 71 m	UC	18
	Kilifi [6]	1994	6 - 71 m	UC	38
	Kilifi [6]	1995	6 - 71 m	UC	29
	Kilifi [6]	1997	3 - 71 m	UC	16
	Kilifi [6]	1998	UK	UK	26
	Kilifi [6]	1999	UK	UK	31
	Kilifi [6]	2000	UK	UK	27
	Kilifi [6]	2001	UK	UK	36
	Kilifi [6]	2002	< 12 y	UC	30
	Kilifi [6]	2003	< 12 y	UC	23
	Kilifi [6]	2006	6 m - 10 y	UC	42
	Siaya [14]	2003	4 - 60 m	UC	75
	Kisii [15]	2005	UK	UC	100
	Kisii [15]	2005	6 - 14 y	AS	100
	Kombewa [15]	2005	UK	UC	100
	Kombewa [15]	2005	6 - 14 y	AS	100
	Kakamega [15]	2005	UK	UC	101
	Kakamega [15]	2005	6 - 14 y	AS	100
	Kakamega [16]	2006	6 - 14 y	AS	84
	Chemelil [16]	2006	6 - 14 y	AS	102
	Kisian [16]	2006	6 - 14 y	AS	105
	Miwani [16]	2006	6 - 14 y	AS	92
	Bondo [17]	2007	6 - 59 m	UC	101
Malawi	Blantyre [4]	1992	children	SM	39
	Blantyre [4]	1993-1994	children	SM	22
	Blantyre [4]	1995-1996	children	SM	24
	Blantyre [4]	1998-1999	children	UC	46
	Blantyre [4]	2000	children	UC	75
	Blantyre [18]	2003-2005	12 - 60 m	UC	244
	Blantyre [5]	2005	6 - 12 y	UC	199
	Blantyre [19]	2005	UK	UK	76
	Blantyre [20]	2007	< 5 y	UC	148
	Salima [21]	1998	< 5 y	UC	72
	Salima [21]	2000	6 - 15 y	AS	137
	Dedza [19]	2005	UK	UK	51
	Mzimba [19]	2005	UK	UK	51
	Karonga [20]	2007	UK	UK	182
Tanzania	Kibaha [22]	1998	young children	UC	51
	Masasi [23]	1999	6 - 60 m	UC	71
	Iringa [24]	2002	UK	UK	17
	Muheza [25]	2003/2004	4 - 59 m	UC	236
	Bagamoyo [26]	2004	children> 6 m	UC	102
	Mbeya [27]	2004/2005	8 m - 55 y	UC	86
	Korogwe [28]	2003	< 20 y	AS	155
	Korogwe [28]	2004	< 20 y	AS	163
	Korogwe [28]	2006	< 20 y	AS	74
	Korogwe [28]	2007	< 20 y	AS	73
Uganda	Kampala [29]	1998-1999	> 6 m	UC	114
	Kampala [30]	1999	adults	UC	32
	Kampala [31]	2004-2007	1 - 10 y	AS	90
	Apac [30]	1999	6 - 59 m	UC	41
	Tororo [30]	1999	6 - 59 m	UC	45
	Tororo [32]	2002-2004	> 6 m	UC	80
	Arua [32]	2002-2004	> 6 m	UC	80
	Mubende [32]	2002-2004	> 6 m	UC	80
	Jinja [32]	2002-2004	> 6 m	UC	80
	Kanungu [32]	2002-2004	> 6 m	UC	80

Country	Site	Year(s)	Age range	Malaria status	Sample size
**West Africa**					
Burkina Faso	Lena [33]	1998	6 m - 15 y	UC	52
	Lena [33]	2001	6 m - 15 y	UC	73
	Bama [33]	1998	6 m - 15 y	UC	73
	Bama [33]	2001	6 m - 15 y	UC	103
	Toussiana [33]	1998	6 m - 15 y	UC	112
	Toussiana [33]	2001	6 m - 15 y	UC	94
	Bobo Dioulasso [33]	1998	6 m - 15 y	UC	103
	Bobo Dioulasso [33]	2001	6 m - 15 y	UC	106
	Bobo Dioulasso [34]	2004	> 6 m	UC	110
	Bourasso [35]	2000	all ages	UC+AS	210
	Ziniare/Bousse [36]	2002	6 - 59 m	UC	999
	Ziniare/Bousse [36]	2002	6 - 59 m	AS	197
	Nouna [37]	2003	6 - 59 m	UC	222
	Nouna [38]	2005	6 - 59 m	UC	109
Guinea-Bissau	Placc/Nhacra [39]	1992	7 - 14 y	AS	31
	Placc/Nhacra [39]	1993	7 - 14 y	AS	18
	Placc/Nhacra [39]	1995	7 - 14 y	AS	49
	Placc/Nhacra [39]	2004	7 - 14 y	AS	93
	Placc/Nhacra [39]	2005	7 - 14 y	AS	23
	Bandim [40]	2001-2004	< 15 y	UC	478
	Bandim [41]	2003	3 - 12 y	UC	333
	Bandim [41]	2004	3 - 12 y	UC	158
	Bandim [41]	2005	3 - 12 y	UC	171
	Bandim [41]	2006	3 - 12 y	UC	115
	Bandim [41]	2007	3 - 12 y	UC	108
Mali	Bandiagara [42]	1998	UK	UC	270
	Kolle [42]	1998	UK	UC	229
	Kolle [42]	1999	UK	UC	294
	Mopti [43]	1997	> 2 y	UC	29
	Bandiagara [3]	1997	> 2 y	UC	62
	Kidal [43]	1999	all ages	SI	56
	Bougoula-Hameau [44]	2002-2004	> 6 m	UC	67
	Bancoumana [45]	1999	UK	UC	93
	Kolle [45,46]	2002	UK/6-59 m	UC	244
	Kolle [45,46]	2003	UK/6-59 m	UC	209
	Bandiagara [45]	2002	UK	UC	152
	Bandiagara [45]	2003	UK	UC	124
	Faladje [45]	2002	UK	UC	18
	Koulikoro Ba [45]	2004	UK	UC	96
	Sirakoro-meg [45]	2004	UK	UC	84
	Niena [45]	2004	UK	UC	34
	Kolébougou [45]	2004	UK	UC	67
	Markakoungo [45]	2004	UK	UC	94
	Dimbal [45]	2004	UK	UC	64
	Kafana [45]	2004	UK	UC	60
	Siékorolé [45]	2004	UK	UC	59
	Toguel [45]	2004	UK	UC	15
	M'pessoba [45]	2004	UK	UC	53
	Banamba [45]	2004	UK	UC	74
	N'debougou [45]	2004	UK	UC	54
Niger	Niamey [47]	2003	3 - 60 m	UC	116
	Niamey [48]	2004	UK	UK	258
	Zindarou/Banizoumbou [8]	2003	all ages	AS	45
	Zindarou/Banizoumbou [8]	2004	all ages	AS	162
	Zindarou/Banizoumbou [8]	2005	all ages	AS	20
	Zindarou/Banizoumbou [8]	2006	all ages	AS	91
